# Comprehensive mitochondrial genomics of *Fasciola gigantica* from Sudan: insights into genetic diversity, evolutionary dynamics, and host adaptation

**DOI:** 10.3389/fvets.2025.1577469

**Published:** 2025-05-01

**Authors:** Bashir Salim, Nouh S. Mohamed, Kamal Ibrahim, Saeed Alasmari, Elisha Chatanga, Yuma Ohari, Nariaki Nonaka, Mohammad A. Alsaad, Faisal Almathen, Ryo Nakao

**Affiliations:** ^1^Camel Research Center, King Faisal University, Al-Hasa, Saudi Arabia; ^2^Molecular Biology Unit, Sirius Training and Research Center, Khartoum, Sudan; ^3^Department of Parasitology, Central Veterinary Research Laboratory, Khartoum, Sudan; ^4^Department of Biology, Faculty of Science and Arts, Najran University, Najran, Saudi Arabia; ^5^Laboratory of Parasitology, Graduate School of Infectious Diseases, Faculty of Veterinary Medicine, Hokkaido University, Sapporo, Japan; ^6^Department of Veterinary Pathobiology, Faculty of Veterinary Medicine, Lilongwe University of Agriculture and Natural Resources, Lilongwe, Malawi; ^7^Department of Microbiology and Parasitology, College of Medicine, Umm AL Qura University, Makkah, Saudi Arabia; ^8^Department of Public Health and Animal Husbandry, College of Veterinary Medicine, King Faisal University, Al-Ahsa, Saudi Arabia; ^9^Division of Parasitology, Veterinary Research Unit, International Institute for Zoonosis Control, Hokkaido University, Sapporo, Japan; ^10^One Health Research Center, Hokkaido University, Sapporo, Japan

**Keywords:** *Fasciola gigantica*, mitochondrial genomics, genetic diversity, phylogenetics, host adaptation

## Abstract

**Introduction:**

This study presents a comprehensive analysis of the complete mitochondrial genomes of *Fasciola gigantica* isolated from cattle, sheep, and goats in Sudan, aiming to provide new insights into genetic diversity, evolutionary dynamics, and host adaptation.

**Methods:**

Mitochondrial genomes were sequenced using high-throughput Illumina MiSeq technology, yielding sequences of 14,483 bp, slightly longer than the reference genome (14,478 bp). A sliding window analysis was conducted to assess nucleotide diversity, and phylogenetic analyses were performed using complete mitochondrial sequences, including and excluding non-coding regions.

**Results:**

Key genetic variations were observed, including a non-canonical start codon (GTG) in the ND5 gene and an alternative stop codon (TAA) in ND4. Length polymorphisms in ND4L and *cox1* suggested potential mitochondrial efficiency adaptations. Non-coding regions showed minor length differences, with the long non-coding region extending by 20 bp and the short by 4 bp. Sliding window analysis identified ND4 and ND5 as the most variable genes, while *cox1*, *nd1,andcox2* were the most conserved. Phylogenetic analysis showed distinct clustering of Sudanese *F. gigantica* isolates with strong bootstrap support. Excluding the D-loop preserved phylogenetic structure, while D-loop-specific analysis revealed high variability, particularly in the sheep isolate.

**Discussion:**

These findings highlight significant genetic variation and evolutionary divergence among *F. gigantica* isolates in Sudan. The observed diversity, particularly within non-coding and variable coding regions, underscores the influence of regional evolutionary pressures and host-associated adaptations. This work enhances understanding of *F. gigantica*’s genetic landscape and supports the development of more targeted molecular surveillance and control strategies for fascioliasis in endemic regions.

## 1 Introduction

Fascioliasis, caused by trematodes of the genus *Fasciola*, is a globally significant yet neglected foodborne parasitic disease affecting both humans and livestock (1). Infections with *Fasciola hepatica* and *Fasciola gigantica* are prevalent among more than 600 million domestic ruminants, including cattle, sheep, goats, buffalo, donkeys, and pigs, leading to severe economic losses estimated at approximately USD 3 billion annually (2). Given its zoonotic potential and substantial impact on livestock production, fascioliasis remains a major concern for veterinary and public health.

Mitochondrial genome sequencing has emerged as a powerful tool for resolving phylogenetic relationships, population genetics, and evolutionary dynamics in parasitic trematodes. The conserved structure and maternal inheritance of mitochondrial DNA make it an ideal molecular marker for species identification and evolutionary studies (3–5). Advances in next-generation sequencing (NGS) technologies have accelerated genomic research on trematodes, leading to the assembly of whole-genome sequences for *F. hepatica* (6), *F. gigantica* (7), and *Paragonimus westermani* (8). These genomic resources have enhanced comparative phylogenetic studies across major trematode taxa, providing deeper insights into parasite evolution and host adaptation.

Genomic evidence suggests that *F. gigantica* and *F. hepatica* diverged approximately 11.8 million years ago (7). The evolutionary expansion of genes related to actin and aquaporin functions has been implicated in the adaptive success of *Fasciola* species, contributing to their large body size, parasitic resilience, and broad geographic distribution.

Recent studies have provided insights into the genetic structure of *F. gigantica* populations in Sudan. Notably, Ibrahim et al. (9) demonstrated that *F. gigantica* in Sudan and other African countries exhibit both host-specific and country-specific lineages based on Maximum Likelihood (ML) phylogenetic trees and median networks. Their findings also suggested that *F. gigantica* infecting small ruminants are evolutionarily distinct from those infecting larger hosts, indicating deep and historical interspecies adaptation. However, their study was limited to partial mitochondrial genes (COI and ND1), leaving gaps in our understanding of the complete mitochondrial genome and its role in shaping evolutionary trajectories.

Mitochondrial genomes typically include a non-coding region often referred to as the AT-rich region, control region, or D-loop—which contains initiation sites for replication and transcription (10). In vertebrates, this non-coding region is known as the D-loop, while in other taxa, it is referred to as the A + T-rich or control region. Mitochondrial genomes, reflecting their eubacterial ancestry, differ from nuclear genomes in codon usage. While ATG is the primary start codon, invertebrates may also use ATT, ATA, or GTG for translation initiation (3, 26). Although gene order, especially among protein-coding and ribosomal RNA genes, has been considered conserved within animal phyla (3), studies on invertebrates such as molluscs, arthropods, flatworms, and nematodes have revealed notable exceptions to this pattern (11–13).

In most animals, mitochondrial tRNAs form a cloverleaf structure, except for tRNA-Ser, which replaces the D-arm with a loop. This distinctive structure was thought to be ancestral to all multicellular animals (3, 26). However, some species also exhibit tRNAs lacking either a D-arm or a T-arm (12, 14). In addition to D-loop variability, structural adaptations in mitochondrial tRNA genes also provide valuable insights into evolutionary dynamics. For example, previous studies on *F. hepatica* have reported the presence of a dihydrouridine arm replacement loop in mitochondrial tRNA-Ser (AGN) (15). This structural modification, observed across metazoans, is considered an early evolutionary adaptation that likely enhances mitochondrial translation efficiency. It replaces the typical D-arm found in standard tRNAs and is considered an early evolutionary adaptation among platyhelminths. The conservation of this feature in trematodes suggests a functional significance that extends beyond structural variation. The presence of this replacement loop may influence tRNA stability and its interaction with the mitochondrial translation machinery, potentially affecting the efficiency of protein synthesis. Understanding these structural nuances provides a more comprehensive view of the molecular evolution and functional adaptations in *F. gigantica*, complementing the findings derived from D-loop variability (16).

Despite recent progress, there remains a critical gap in understanding the complete mitochondrial genome architecture of *F. gigantica* in Sudan, especially its potential role in host adaptation and population structure.

Building on these findings, the present study aims to provide a more comprehensive genomic perspective by analyzing the complete mitochondrial genome of *F. gigantica* from Sudanese livestock. By integrating whole-genome mitochondrial data, we seek to refine phylogenetic relationships, elucidate genetic diversity, and assess evolutionary adaptations across different hosts, thereby advancing our knowledge of *F. gigantica* population dynamics in Sudan and beyond.

## 2 Materials and methods

### 2.1 Sample collection and DNA extraction

Sample collection and DNA extraction were conducted following the protocols outlined by Ibrahim et al. (9). A total of 16 samples were collected: ten from cattle, five from goats, and one from sheep, aimed at generating complete mitochondrial genome sequences. Samples were sourced from slaughterhouses, thoroughly washed with distilled water, and identified morphologically. To facilitate genomic DNA extraction, the samples were squashed, and approximately 10 mg of tissue from the lateral zone was used. DNA extraction was carried out using the QIAamp DNA Extraction Kit (Qiagen, Inc.), following the manufacturer’s guidelines.

### 2.2 PCR and Illumina MiSeq sequencing

To obtain the complete mitochondrial genome sequences, two overlapping long PCR reactions were performed using newly designed primers in this study. The first set, using forward primer P101 (5′-TCTAGTGCTGCTTTGGTTGCTATGTC-3′) and reverse primer P265 (5′-GAAGCTACAATCCCACGCACTAAACT-3′), amplified a fragment of approximately 7 kb. The second set, with forward primer P247 (5′-TGTGGGTTATTGTACGGAGTTGTGTG-3′) and reverse primer P121 (5′-CATATAACCCAACACCTTACGCTCACC-3′), amplified a fragment of approximately 9 kb. Each PCR reaction was performed in a 25 μl reaction mixture containing 12.5 μl of 2 × Gflex PCR Buffer (Mg^2+,^ dNTP plus), 0.5 μl of Tks Gflex DNA Polymerase (1.25 units/μl, TaKaRa Bio Inc., Shiga, Japan), 200 nM of each primer, 1.0 μl of template DNA, and molecular-grade water. The cycling conditions included an initial denaturation at 94°C for 1 min, followed by 35 cycles of denaturation at 98°C for 10 s, annealing at 60°C for 15 s, extension at 68°C for 7 min, and a final extension at 68°C for 5 min. The PCR products were then electrophoresed on a 1% agarose gel stained with Gel-Red (Biotium, Hayward, CA, USA) and visualized under UV light.

Following PCR amplification, the amplicons were purified using the NucleoSpin Gel and PCR Clean-Up Kit (TaKaRa Bio Inc.). The purified amplicons were subsequently sequenced on an Illumina MiSeq platform using the MiSeq Reagent Kit v3 (600 cycles) (Illumina, San Diego, USA), following the manufacturer’s protocol. The reads obtained from the Illumina MiSeq run were mapped against published mitochondrial genomes of *F. gigantica* (NC_024025). Liu et al. (17) using CLC Genomics Workbench v20.0.4 (QIAGEN, Hilden, Germany). The sequences generated in this study have been deposited in the NCBI GenBank database under accession numbers LC649568–LC649583.

### 2.3 Mitochondrial genome organization

The complete mitochondrial genome of *F. gigantica* was compared with the reference sequence for *F. gigantica*, NC_024025. Liu et al. (17) to analyze gene content, gene order, strand orientation, and tRNA arrangements. Circular genome maps were generated using the online tool GenomeVx software,^1^ which allows for the input of gene coordinates and strand direction to produce high-quality, customizable graphical representations. This visualization facilitated a clear comparison of coding regions (protein-coding genes and rRNAs) and non-coding elements, including tRNAs and the control region. In addition, the use of this platform enabled the identification of structural features such as strand bias and the relative positioning of genes, which are essential for interpreting evolutionary patterns and genome architecture in *F. gigantica*.

### 2.4 Sliding window analysis of nucleotide variation

To identify variable nucleotide sites, pairwise alignments of the 16 complete mitochondrial genomes of *F. gigantica* from Sudan were conducted using MUSCLE, implemented in MEGA 10. Sliding window analysis of the aligned mitochondrial genome sequences was then performed using DnaSP v.5. A sliding window of 300 bp, with a 10 bp overlap between steps, was employed to estimate nucleotide diversity (*π*) across the alignment. The nucleotide diversity was plotted against the mid-point positions of each window, and gene boundaries were marked to correlate variations with specific genomic regions.

### 2.5 Phylogenetic analysis

#### 2.5.1 Phylogenetic relationship of *F. gigantica* trematodes

The phylogenetic relationships among 16 mitochondrial genomes of *F. gigantica* from Sudan and selected species from the genera *Fasciola, Fascioloides, Fasciolopsis, Echinostoma, Paragonimus, Opisthorchis, Clonorchis, Schistosoma, Trichobilharzia,* and *Orientobilharzia* were analyzed using maximum likelihood methods. The phylogenetic tree was constructed with MEGA software, applying the best-fit substitution model JTT + G + F. This model was selected based on the lowest Bayesian Information Criterion (BIC) score using MEGA’s built-in model selection tool, ensuring the most appropriate fit for the data. The tree was subsequently visualized using the ggplot2 package in R (v3.6.0). *Gyrodactylus derjavinoides* (GenBank accession number NC_010976) was used as an outgroup to root the tree and clarify evolutionary relationships.

#### 2.5.2 Phylogenetic analysis using complete mitochondrial genomes, excluding and including the stem-loop (D-Loop) non-coding region

The stem-loop phylogenetic relationships among *F. gigantica samples* from cattle, goats, and sheep were inferred using sequences from the complete mitochondrial genome, the mitochondrial genome excluding the D-Loop, and the D-Loop region alone. Additionally, the stem-loop structure, referred to as the AT-Loop in the reference sequence (NC_024025), was analyzed separately for the 16 sequences to assess its role in genetic variability and diversity. Three different scenarios were employed to construct ML trees based on nucleotide sequences to explore the evolutionary significance of different regions of the mitochondrial genome in *F. gigantica*: Complete mitochondrial genome (i) The entire mitochondrial genome sequences were used to construct the ML tree, (ii) Mitochondrial genome excluding the D-Loop: The D-Loop region was excluded from the analysis, and the remaining mitochondrial genome sequences were used to construct the ML tree, and (iii) D-Loop region only: The D-Loop region sequences alone were used to construct the ML tree. Sequences were aligned using the MUSCLE algorithm implemented in MEGA version 10. The best-fit substitution models were determined using the Bayesian Information Criterion (BIC) for each dataset. The HKY + G + I model was selected for both the complete mitochondrial genome and the mitochondrial genome excluding the D-Loop. The HKY model was applied for the D-Loop region alone.

ML phylogenetic trees were constructed using these models, with bootstrap analysis performed with 1000 replicates to assess the statistical support for the branches. The final topologies and branch supports were visualized using FigTree version 1.4.4 (18). This approach allowed for a comprehensive comparison of the evolutionary relationships inferred from different regions of the mitochondrial genome, providing insights into the genetic variability and potential host-specific adaptations in *F. gigantica* from Sudan.

## 3 Results and discussion

### 3.1 Genome content and organization

**Figure 1:**
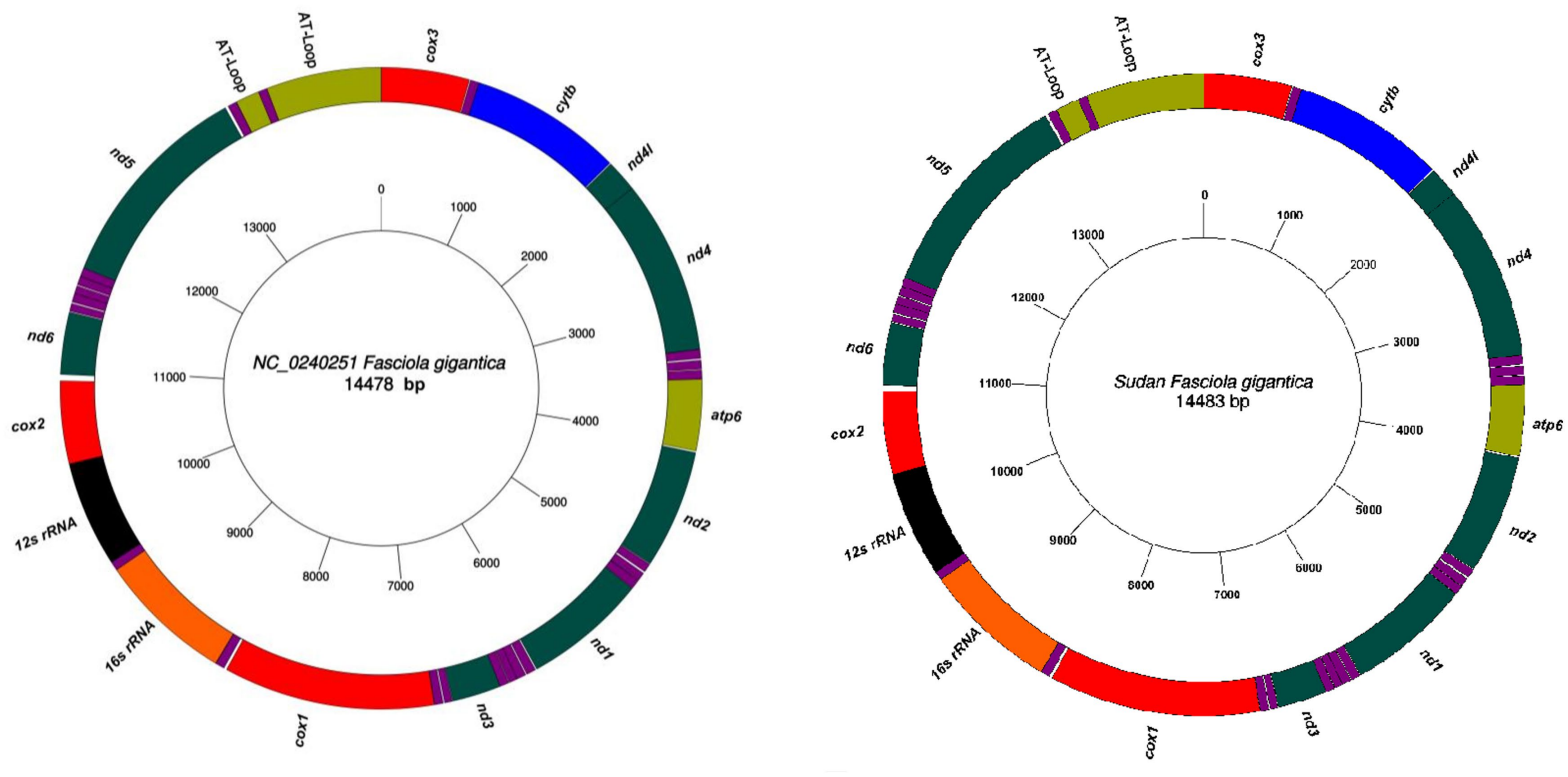
The complete mitochondrial genome sequences of *F. gigantica* obtained from Sudan with a length of 14,483 bp. All genes have standard nomenclature including the 22 tRNA genes, which are designated purple color. Comparison with the reference sequence (NC_024025) revealed an additional 5 bp in the Sudanese isolates, resulting in the observed genome length. Gene locations are detailed in Tables 1 and 2.

Sixteen complete mitochondrial genome sequences of *F. gigantica* were successfully obtained from cattle, sheep, and goats, each with a length of 14,483 bp (Figure 1). The mitochondrial genome of *F. gigantica* comprises 12 protein-coding genes (*cox1-3, nd1-6, nd4l, atp6,* and *cytb*), a small subunit ribosomal RNA gene (rrnS), a large subunit ribosomal RNA gene (rrnL), 22 transfer RNA genes, and both long and short non-coding regions (Table 1). Detailed gene locations are provided in Table 2. A comparison with the reference sequence revealed an additional 5 bp in the mitochondrial genomes from Sudan, resulting in a total length of 14,483 bp, compared to the reference sequence’s 14,478 bp (see Figure 1).

**Table 1:** Comparison of the lengths, start codons, and stop codons of protein-coding genes in the mitochondrial genome of *F. gigantica* from Sudan with the Reference genome.

Genes/Proteins	Length (bp)	Length (aa)	Codon Start	Codon Stop	Position (5′ to 3′)
COX3					
Sudan	642	214	ATG	TAG	1–642
Reference	642	213	ATG	TAG	1–642
CYTB					
Sudan	1,113	371	ATG	TAG	715–1827
Reference	1,113	370	ATG	TAG	715–1827
ND4L					
Sudan	261	87	GTG	TAG	1,836–2096
Reference	273	90	GTG	TAG	1,836–2,108
ND4					
Sudan	1,272	424	GTG	TAG	2,057–3,328
Reference	1,272	424	GTG	TAA	2,069–3,337
ATP6					
Sudan	519	173	ATG	TAG	3,545–4,063
Reference	519	172	ATG	TAG	3,554–4,072
ND2					
Sudan	867	289	ATG	TAG	4,076–4,942
Reference	867	288	ATG	TAG	4,085–4,951
ND1					
Sudan	903	301	GTG	TAG	5,162–6,064
Reference	903	300	GTG	TAG	5,171–6,073
ND3					
Sudan	357	119	GTG	TAG	6,342–6,698
Reference	357	118	GTG	TAG	6,364–6,720
COX1					
Sudan	1,541	514	GTG	TAG	6,834–8,375
Reference	1,533	510	GTG	TAG	6,856–8,397
COX2					
Sudan	603	201	ATG	TAG	10,287–10,889
Reference	603	200	ATG	TAG	10,310–10,912
ND6					
Sudan	453	151	ATG	TAG	10,936–11,388
Reference	453	150	ATG	TAG	10,959–11,411
ND5					
Sudan	1,569	523	ATG	TAG	11,724–13,292
Reference	1,569	522	GTG	TAG	11,744–13,309

**Table 2:** Comparison of lengths and positions of transfer RNAs, ribosomal RNAs, and non-coding regions in the mitochondrial genome of *F. gigentica* from Sudan with the reference genome.

Gene (RNA)	Length (bp) Sudan	Position 5′ to 3′ Sudan	Position 5′ to 3′ Reference	Length (bp) Reference
tRNA-His	62	650–713	650–713	64
tRNA-Gln	64	3,330–3,395	3,339–3,404	66
tRNA-Phe	63	3,408–3,472	3,417–3,481	65
tRNA-Met	64	3,479–3,544	3,488–3,553	66
tRNA-Val	62	4,948–5,011	4,957–5,020	64
tRNA-Ala	63	5,026–5,090	5,035–5,099	65
tRNA-Asp	63	5,094–5,158	5,103–5,199	65
tRNA-Asn	66	6,070–6,137	6,084–6,153	70
tRNA-Pro	66	6,143–6,210	6,163–6,253	68
tRNA-Ile	59	6,211–6,271	6,231–6,292	62
tRNA-Lys	64	6,276–6,341	6,297–6,363	67
tRNA-Ser	54	6,703–6,758	6,725–6,780	56
tRNA-Trp	61	6,768–6,830	6,790–6,852	63
tRNA-Thr	66	8,397–8,464	8,419–8,486	68
L-rRNA (16S)	**984**	**8,466–9,451**	8,488–9,473	986
tRNA-Cys	63	9,452–9,516	9,474–9,538	65
S-rRNA (12S)	**768**	**9,517–10,286**	9,539–10,309	771
tRNA-Tyr	55	11,396–11,452	11,419–11,475	57
tRNA-Leu	63	11,463–11,527	11,486–11,550	65
tRNA-Ser	57	11,528–11,586	11,551–11,607	57
tRNA-Leu	62	11,595–11,658	11,616–11,678	63
tRNA-Arg	67	11,660–11,728	11,680–11,745	66
tRNA-Glu	66	13,315–13,382	13,332–13,399	68
tRNA-Gly	62	13,559–13,622	13,574–13,637	64
Non-coding region				
Short NR	176	13,383–13,621	13,400–13,573	174
Long NR	860	13,623–14,483	13,638–14,478	841

Bolded values indicate the mitochondrial 12S and 16S rRNA genes, highlighted due to their conserved structure and relevance in phylogenetic and evolutionary analyses.

The successful sequencing of the 16 complete mitochondrial genomes of *F. gigantica* from Sudan reveals a consistent genetic organization with a slight increase in genome length compared to the reference sequence (NC_024025). The additional 5 bp observed in the Sudanese isolates suggests minor insertions that could have occurred due to evolutionary processes specific to these populations. Although the overall structure of the mitochondrial genome remains highly conserved, reflecting its critical role in cellular metabolism and energy production, this variation in genome length may have functional implications, particularly if the insertions are located in regulatory or non-coding regions that influence gene expression.

The mitochondrial genome organization observed in *F. gigantica* comprising 12 protein-coding genes, two ribosomal RNA genes, 22 transfer RNA genes, and non-coding regions—is typical of trematodes, indicating a high degree of conservation across related species. However, the presence of these slight differences in genome length between the Sudanese isolates and the reference sequence could be indicative of local adaptation or genetic drift. These findings emphasize the importance of investigating genetic variability within and between populations of *F. gigantica*, as even minor variations can have significant biological implications. Moreover, the detailed mapping of gene locations confirms that the overall mitochondrial genome architecture is maintained, with the observed length difference likely arising from localized genetic events rather than large-scale rearrangements. Further research is needed to explore the potential impact of these variations on the parasite’s lifecycle, host interactions, and adaptability to diverse environmental conditions. Understanding these genetic differences could provide valuable insights into the evolution and ecology of *F. gigantica*, particularly in the context of developing targeted control strategies.

The observed start and stop codon variations may have functional consequences for mitochondrial gene expression and translation efficiency. The presence of a non-canonical start codon (GTG) in ND5, while rare, has been reported in other invertebrate mitochondrial genomes and may indicate adaptations in mitochondrial initiation machinery (19, 20). Similarly, the use of an alternative stop codon (TAA) in ND4 could reflect translational flexibility or regulatory divergence, potentially linked to selective pressures in the parasite’s lifecycle or host environment.^2^ Moreover, the length polymorphisms in ND4L and COX1 may affect protein structure or stability, potentially influencing electron transport chain performance, which is critical for energy metabolism and survival in varying host conditions (21). These changes, along with expansions in the non-coding regions, may collectively represent fine-tuned regulatory adaptations shaped by local environmental or host-specific factors in Sudanese isolates (22, 23).

The observed mitochondrial gene variations in *F. gigantica* could significantly influence parasite metabolism, host adaptation, and potentially drug resistance. Non-canonical start codons and alternative stop codons may impact mitochondrial protein synthesis, possibly altering translation efficiency and downstream energy metabolism factors that can affect parasite fitness and adaptability to different host environments (20, 22). Length polymorphisms in genes such as *nd4l* and *cox1*, which are involved in the mitochondrial electron transport chain, may influence ATP production and oxidative phosphorylation, thereby contributing to variation in metabolic efficiency and survival across host species (21, 23). Expansions in non-coding regions, including the D-loop, may affect the regulation of replication and transcription, providing a mechanism for adaptive gene regulation in response to host-or environment-specific pressures (22). Comparable mitochondrial variations in *F. hepatica* have been associated with broader host range and geographic adaptability, suggesting a role for mitogenomic flexibility in supporting ecological plasticity (24). Furthermore, genetic variation has been linked to reduced drug sensitivity in trematodes. For instance, in *Schistosoma mansoni*, mutations in a transient receptor potential (TRP) channel have been shown to mediate praziquantel response (25), highlighting how genomic changes potentially including mitochondrial elements can influence treatment outcomes. Together, these findings underscore the importance of mitochondrial genome evolution not only in understanding parasite adaptation and host specificity, but also in anticipating potential drug resistance mechanisms in *F. gigantica*.

### 3.2 Protein-coding genes and codon usage patterns

The boundaries of protein-coding genes in the mitochondrial genome of Sudanese *F. gigantica* were determined by aligning the sequences and identifying the translation initiation and termination codons, compared to the reference sequence (NC_024025). As shown in Table 1, the start codon for 11 of the 12 protein-coding genes was ATG, consistent with the reference mitochondrial genome, except for ND5, which used a GTG start codon. Similarly, the stop codon was TAG for most genes, consistent with the reference sequence, except for ND4, which used a TAA stop codon.

In terms of gene length, 10 of the 12 protein-coding genes were consistent with the reference, while differences were observed in ND4L and COX1. Specifically, ND4L in the reference sequence was 12 bp longer at 273 bp, compared to 261 bp in the Sudanese isolates. Conversely, COX1 in the reference was 9 bp shorter at 1,533 bp, compared to 1,542 bp in the Sudanese isolates. Additionally, all amino acid sequences differed from those in the reference mitochondrial genome, indicating variations in codon usage patterns.

The results demonstrate that the majority of the protein-coding genes in the mitochondrial genome of Sudanese *F. gigantica* exhibit conserved start and stop codons, consistent with the reference genome (NC_024025). The exception in the start codon for ND5 (GTG instead of ATG) and the stop codon for ND4 (TAA instead of TAG) suggests slight deviations in the genetic code that could influence the translation process, potentially affecting protein synthesis efficiency or function in these populations. These variations, though subtle, might reflect local adaptations or evolutionary pressures specific to the Sudanese *F. gigantica* populations. The differences observed in the lengths of ND4L and COX1 between the Sudanese isolates and the reference sequence further indicate genetic variability within the species. The 12 bp reduction in ND4L and the 9 bp increase in COX1 may impact the structure and function of the resulting proteins, potentially altering the parasite’s physiology or its ability to adapt to different environmental conditions or hosts. Moreover, the observed differences in amino acid sequences suggest that the Sudanese populations of *F. gigantica* may have distinct codon usage patterns compared to the reference sequence. These variations could result from selective pressures that favor certain codons over others, possibly due to differences in the availability of tRNAs or the metabolic demands of the parasite in different hosts. Such variations in codon usage may also contribute to the overall genetic diversity of *F. gigantica*, affecting its evolutionary trajectory and adaptability. These findings highlight the importance of understanding the genetic diversity within parasitic species, as even minor differences in protein-coding genes and codon usage can have significant biological implications. Further research could explore the functional consequences of these genetic variations and their potential impact on the parasite’s lifecycle, virulence, and interaction with hosts.

### 3.3 Transfer RNA and ribosomal RNA genes and non-coding regions

The mitochondrial genome of *F. gigantica* contains 22 tRNA genes, with lengths ranging from 56 to 69 nucleotides, as detailed in Table 2. The order and orientation of these tRNA genes were consistent with the reference sequence, indicating a conserved gene arrangement pattern. However, differences were observed in the lengths of the non-coding regions. Specifically, the long non-coding region in the Sudanese isolates was 20 bp longer, measuring 861 bp compared to 841 bp in the reference sequence. Similarly, the short non-coding region was 4 bp longer in the Sudanese isolates, at 178 bp compared to 174 bp in the reference sequence.

The consistency in the order and orientation of the 22 tRNA genes between the Sudanese isolates and the reference sequence underscores the conserved nature of the mitochondrial genome within *F. gigantica*. This conservation is expected given the critical roles that tRNA genes play in protein synthesis, where even minor alterations could potentially disrupt essential cellular functions. The uniformity in tRNA gene arrangement further supports the notion that mitochondrial genomes are generally stable, particularly in their coding regions, due to their essential role in maintaining the parasite’s metabolic functions. However, the observed differences in the lengths of the non-coding regions, particularly the long and short non-coding regions, may be of evolutionary significance. The 20 bp increase in the long non-coding region and the 4 bp increase in the short non-coding region in the Sudanese isolates suggest that these regions are more susceptible to variation. Non-coding regions in mitochondrial DNA often contain regulatory elements crucial for the replication and transcription of the mitochondrial genome. Therefore, these length variations could reflect adaptations to local environmental conditions or host interactions that may have influenced the evolutionary trajectory of *F. gigantica* in Sudan. Moreover, the expansion of non-coding regions could potentially be linked to the regulation of gene expression or the stability of the mitochondrial genome. Variations in non-coding regions might affect the binding of regulatory proteins or the secondary structure of the DNA, which in turn could influence the replication efficiency or the transcriptional activity of the mitochondrial genes. This flexibility in non-coding regions may provide the parasite with an adaptive advantage, allowing it to thrive in diverse environments and host species. In conclusion, while the tRNA and ribosomal RNA genes of *F. gigantica* show a high degree of conservation, the variations in the non-coding regions highlight the dynamic nature of these areas within the mitochondrial genome. These findings contribute to our understanding of the genetic diversity within *F. gigantica* and may offer insights into how these parasites adapt to different ecological niches and hosts.

### 3.4 Nucleotide variability in the mitochondrial genome among *F. gigantica* from Sudan

**Figure 2:**
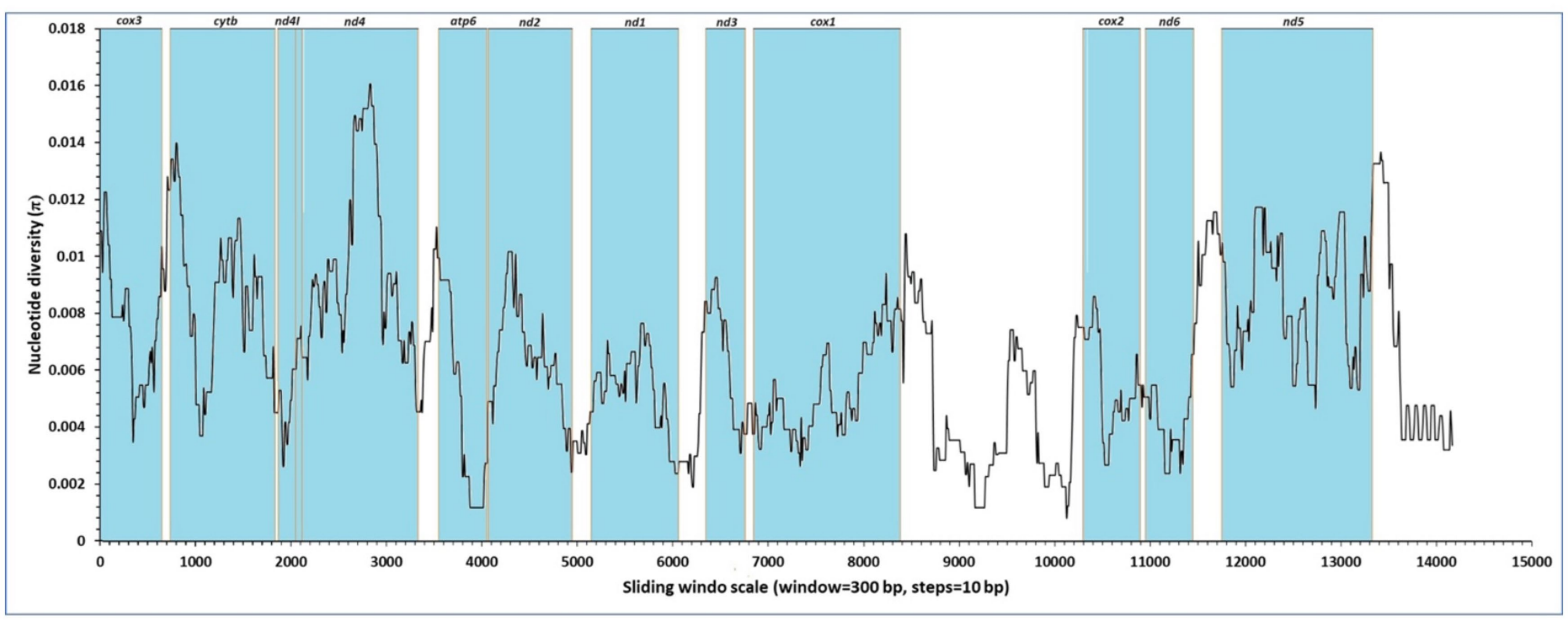
Sliding window analysis of nucleotide diversity (*π*) across the complete mitochondrial genomes of *F. gigantica* from Sudan. The x-axis represents the position along the mitochondrial genome, while the y-axis indicates nucleotide diversity (π). The analysis was performed with a window size of 300 bp and a step size of 10 bp. The regions corresponding to the protein-coding genes (*cox3*, *cytb*, *nd4l*, *nd4*, *atp6*, *nd2*, *nd1*, *nd3*, *cox1*, *cox2*, *nd6*, and *nd5*) are highlighted in blue. The highest nucleotide diversity is observed in the *nd4 and nd5* genes, indicating regions of elevated genetic variability.

Nucleotide diversity (*π*) estimates for individual mitochondrial genes were obtained through sliding window analysis across the mitochondrial genomes of *F. gigantica* (Figure 2). The sliding window analysis revealed variable levels of sequence diversity across different regions of the mitochondrial genome. Specifically, the analysis showed that the *nd4* and *nd5* genes exhibited the highest levels of sequence variability, with prominent peaks in nucleotide diversity. In contrast, the *cox1, nd1,* and *cox2* genes were identified as the most conserved regions, showing lower levels of nucleotide diversity. The analysis also highlighted that the *cox3, cytb, nd4L,* and *atp6* genes showed moderate variability, while the *nd3, nd6,* and *nd2* genes demonstrated relatively low sequence variability. These findings suggest that while certain regions of the mitochondrial genome are highly conserved, others are subject to higher levels of variation, which may be indicative of different selective pressures acting on these genes.

The results from the Sudanese isolates contrast with previous findings from the reference genome (NC_024025), where the *nd6* and *cytb* genes were reported to have the lowest levels of sequence variability, and the *nd1* and *cox1* genes were identified as conserved regions. In this study, however, the *nd4* and *nd5* genes were among the least conserved, highlighting the presence of genetic diversity in these regions within the Sudanese populations of *F. gigantica*.

This analysis underscores the importance of understanding nucleotide variability within the mitochondrial genome, as it provides insights into the evolutionary dynamics and potential adaptive significance of different genomic regions in *F. gigantica*. The observed variations could have implications for the parasite’s adaptation to different hosts or environmental conditions in Sudan.

### 3.5 Phylogenetic analysis of *F. gigantica* and related trematodes

**Figure 3:**
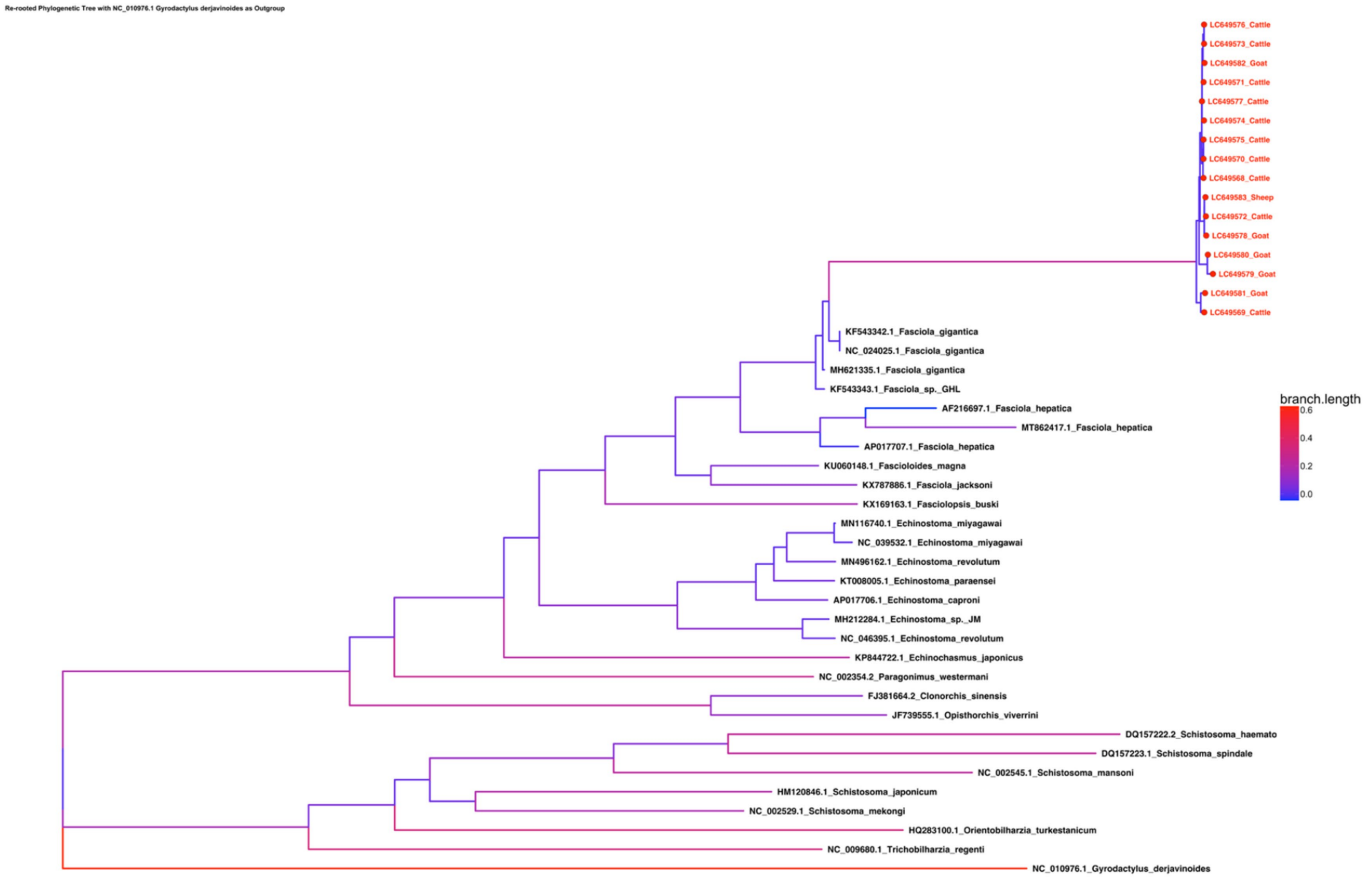
Phylogenetic tree of the concatenated amino acid sequence data for all 12 protein-coding genes of *F. gigantica* isolates from Sudan, alongside selected species from the genera *Fasciola*, *Fascioloides*, *Echinostoma*, *Schistosoma*, *Opisthorchis*, *Clonorchis*, and *Paragonimus*. The tree reveals a well-supported clade of Sudanese *F. gigantica* closely related to other isolates from China and Thailand, with minimal genetic divergence within the Sudanese population. The *Fasciola* genus is shown to be closely related to *Fascioloides* and *Echinostoma*, while *Schistosoma* forms a distinct clade, indicating significant evolutionary distance.

The phylogenetic tree constructed from the complete mitochondrial genomes of 16 *F. gigantica* isolates from Sudan, along with selected species from the genera *Fasciola*, *Fascioloides*, *Echinostoma*, *Schistosoma*, *Opisthorchis*, *Clonorchis*, *Paragonimus*, and others, reveals clear clustering patterns (Figure 3). The *F. gigantica* isolates from Sudan form a well-supported clade, closely related to other *F. gigantica* isolates from different regions, including those from China and Thailand. Within the *Fasciola* genus, *F. gigantica* clusters with *F. hepatica* and *Fasciola jacksoni*, forming a distinct group separate from other trematode species. The *F. gigantica* isolates from Sudan exhibit very little genetic divergence among themselves, as indicated by the short branch lengths, suggesting high genetic similarity within this population. However, there is clear genetic differentiation between *F. gigantica* and other species within the same genus, such as *F. hepatica* and *F. jacksoni*.

The broader phylogenetic tree indicates that the *Fasciola* genus is more closely related to *Fascioloides* and *Echinostoma* species than to the more distantly related Schistosoma species, which form a separate clade. The outgroup, *G. derjavinoides*, is positioned farthest from the other species, as expected, confirming the robustness of the phylogenetic analysis. The phylogenetic analysis confirms that the *F. gigantica* isolates from Sudan are genetically homogeneous and form a distinct clade within the *Fasciola* genus. This clustering indicates that the Sudanese population of *F. gigantica* is relatively isolated with limited genetic diversity, which could be due to the geographical and environmental conditions specific to Sudan. The close clustering of *F. gigantica* isolates with other regional isolates from Asia suggests some level of historical gene flow or a common ancestry among these populations, although the Sudanese isolates still maintain distinct genetic characteristics. The clear genetic differentiation between *F. gigantica* and other species such as *F. hepatica* and *F. jacksoni* highlights the evolutionary divergence within the *Fasciola* genus. The differentiation could be attributed to adaptations to different hosts or environments, which may have driven speciation events within this group of parasites. The broader phylogenetic relationships show that the *Fasciola* genus is closely related to *Fascioloides* and *Echinostoma*, which is consistent with the morphological and biological similarities among these genera. However, the distinct separation of *Schistosoma* species into a separate clade underlines the significant evolutionary distance between these trematodes and those within the *Fasciola* genus.

Overall, this phylogenetic analysis offers valuable insights into the genetic relationships and evolutionary history of *F. gigantica* and related trematode species. The observed homogeneity of among Sudanese isolates may have important implications for control strategies, as reduced genetic diversity could either simplify intervention efforts by targeting a relatively uniform population or complicate them if such uniformity masks cryptic adaptation or drug resistance potential.

This genetic uniformity may be shaped by underlying population dynamics such as recent evolutionary bottlenecks or restricted gene flow, potentially influenced by the limited geographic distribution and genetic similarity of the intermediate snail host, in combination with livestock movement patterns and ecological barriers that constrain parasite dispersal. While this study focused on mitochondrial genome variation, future research should incorporate nuclear genomic data, characterization of local snail host populations, and expanded sampling across neighboring regions to better resolve the evolutionary forces and epidemiological patterns influencing *F. gigantica* diversity in East and Central Africa.

### 3.6 Comparative phylogenetic analysis using complete mitochondrial genomes and D-Loop variability

#### 3.6.1 Complete mitochondrial genome

**Figure 4:**
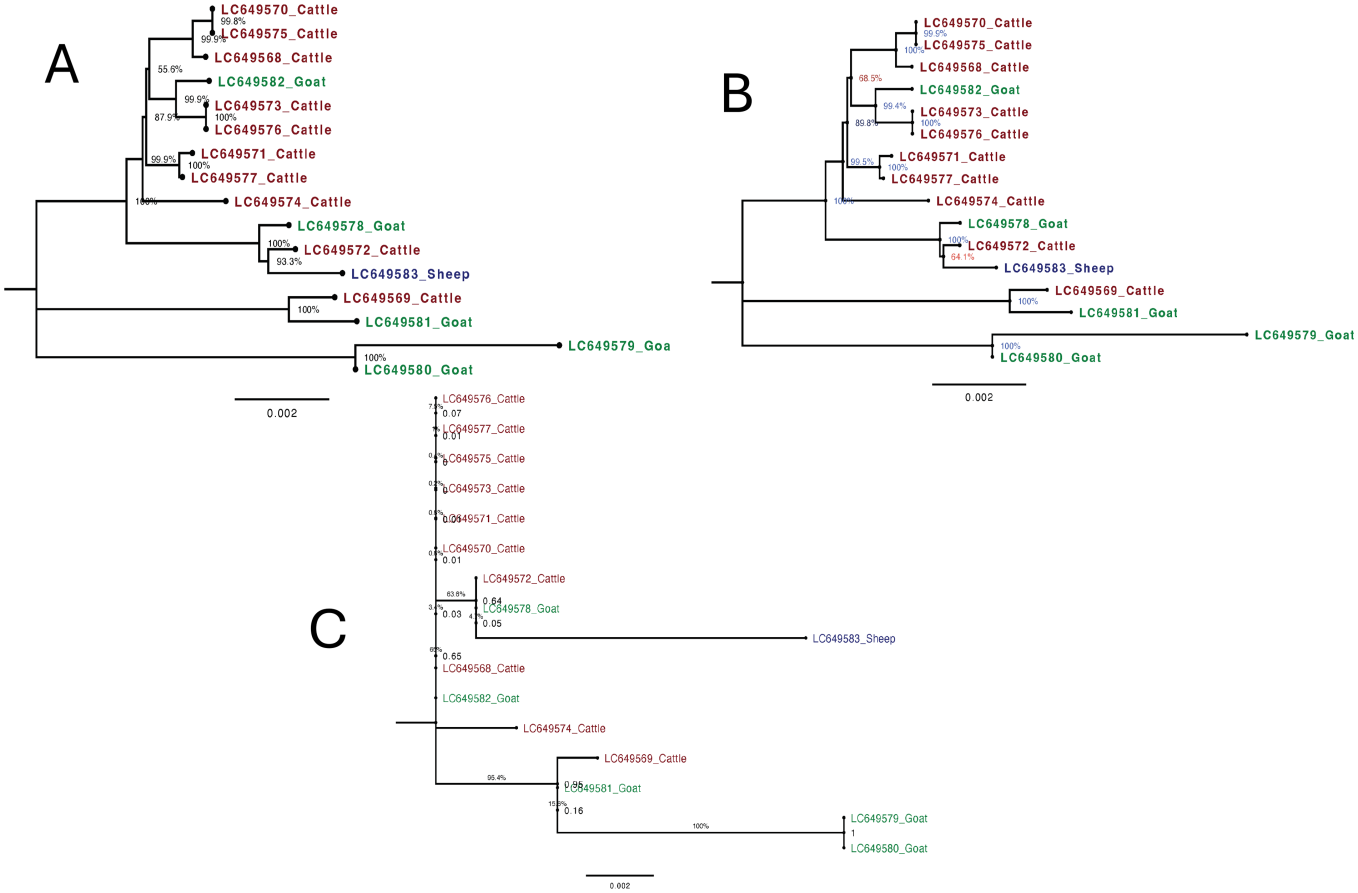
Maximum likelihood trees of *F. gigantica* isolates from Sudan from cattle, goats, and sheep from using different mitochondrial genome regions. **(A)** Phylogenetic tree based on complete mitochondrial genome sequences. The sheep sample (LC649583_Sheep) clusters closely with a cattle sample (LC649572_Cattle) with strong bootstrap support (100%). Goat samples from two distinct clades, with LC649582_Goat showing a closer relationship to some cattle samples, suggesting recent divergence or introgression events. **(B)** Phylogenetic tree excluding the D-loop region. The overall structure is similar to the complete genome tree. The sheep sample (LC649583_Sheep) continues to cluster with the cattle sample (LC649572_Cattle) with strong bootstrap support (100%). Goat samples remain divided into two distinct clades, with LC649582_Goat showing some differentiation, indicating that major evolutionary relationships are preserved even without the D-loop. **(C)** Phylogenetic tree based solely on the D-loop region. The sheep sample (LC649583_Sheep) exhibits a significantly longer branch length, indicating a higher degree of variability. This suggests that the D-loop region captures recent evolutionary changes or host-specific mutations not evident in the complete mitochondrial genome.

The phylogenetic tree constructed using the complete mitochondrial genome sequences of *F. gigantica* from cattle, goats, and sheep revealed distinct clustering patterns with high bootstrap support (Figure 4A). The sheep sample (LC649583_Sheep) clustered closely with a cattle sample (LC649572_Cattle), with a short branch length and strong bootstrap support (100%), indicating a recent shared evolutionary history between these two samples. The goat samples formed two well-supported clades: one group consisted of LC649579_Goat and LC649580_Goat, which clustered distinctly from the rest, while the other group included LC649581_Goat, LC649578_Goat, and LC649582_Goat. The latter sample (LC649582_Goat) showed a closer relationship to some cattle samples, suggesting possible recent divergence or introgression events.

#### 3.6.2 Mitochondrial genome without D-Loop

Excluding the D-loop region, the phylogenetic tree maintained a similar overall structure to the complete genome tree (Figure 4B). The sheep sample (LC649583_Sheep) continued to cluster with the cattle sample (LC649572_Cattle) with strong bootstrap support (100%), and the branch length remained short, reinforcing the recent evolutionary relationship between these samples. The goat samples also remained divided into two distinct groups, with LC649579_Goat and LC649580_Goat forming a separate clade, and LC649581_Goat and LC649578_Goat clustering together, with LC649582_Goat showing some differentiation. The tree demonstrated that the major evolutionary relationships were preserved even without the D-loop, although it might underrepresent some of the recent divergence captured by the complete genome.

#### 3.6.3 D-Loop only

The phylogenetic tree based solely on the D-loop region showed notable differences in branch lengths and clustering patterns (Figure 4C). The sheep sample (LC649583_Sheep) displayed a significantly longer branch length compared to the complete genome and without D-loop trees, indicating a higher degree of variability in this region. This suggests that the D-loop region captures recent, rapid evolutionary changes or host-specific mutations that are not as apparent when using the entire mitochondrial genome. However, this increased variability is accompanied by lower bootstrap support, reflecting greater uncertainty in the relationships inferred from the D-loop alone. Among the goat samples, the same two groups were present, but with less distinct separation and shorter branch lengths compared to the complete genome tree. The clustering of LC649582_Goat closer to cattle samples in the complete genome tree was less pronounced here, indicating that the D-loop region alone might not provide enough resolution to accurately reflect some evolutionary relationships.

The phylogenetic analysis based on the complete mitochondrial genome provided the most reliable and detailed insights into the evolutionary relationships and variability within *F. gigantica* from different host species. The high bootstrap support across the tree reflects strong confidence in the inferred relationships, suggesting that the complete genome captures both recent and ancient evolutionary events effectively. The clustering of the sheep sample with a cattle sample, and the distinct clades within the goat samples, highlight the ability of the complete genome to detect subtle recent divergences and possible host-specific adaptations. The short branch lengths observed in this tree indicate close genetic relationships, supporting the notion of recent divergence.

When the D-loop region was excluded from the analysis, the overall tree topology remained largely unchanged, with major relationships preserved and strong bootstrap support still evident. This indicates that the coding regions of the mitochondrial genome provide a stable and reliable signal for phylogenetic analysis, particularly for understanding deeper evolutionary relationships. The short branch lengths observed for the sheep sample and others reinforce the close evolutionary relationships. However, the absence of the D-loop may underrepresent very recent divergences or host-specific mutations, which are better captured by the complete genome.

In contrast, the phylogenetic tree based solely on the D-loop region revealed more variability and introduced some inconsistencies in the clustering of samples, particularly within the sheep and goat groups. The longer branch length observed for the sheep sample (LC649583_Sheep) in the D-loop tree suggests that this region captures more recent evolutionary events or host-specific variations that are not as detectable when the entire genome is considered. However, the lower bootstrap values across the tree suggest that while the D-loop captures recent evolutionary changes, it does so with less precision and greater uncertainty. This variability may be useful for studying very recent divergence or population-specific mutations, but it also introduces noise that can obscure the broader evolutionary relationships.

#### 3.6.4 Use of the D-Loop as a diagnostic marker

The D-loop region’s high variability makes it a potentially useful diagnostic marker for studying recent evolutionary changes and host specificity in *F. gigantica*. Its ability to capture recent mutations suggests it could be valuable in epidemiological studies aimed at tracking the spread and adaptation of the parasite across different host species. However, due to the noise and lower resolution associated with the D-loop, it should be used in conjunction with more conserved regions of the mitochondrial genome for a more comprehensive understanding. This complementary approach would balance the sensitivity to recent changes with the stability required for accurate phylogenetic inference, making it more effective for both evolutionary studies and epidemiological surveillance.

This study provides a comprehensive analysis of the complete mitochondrial genomes of *F. gigantica* from diverse host species in Sudan, revealing significant insights into the genetic diversity, evolutionary dynamics, and adaptation mechanisms of this neglected parasitic species. The Sudanese isolates displayed minor but notable variations in genome length, codon usage, and non-coding regions compared to the reference genome, indicating potential localized evolutionary adaptations. Phylogenetic analyses underscore the genetic homogeneity of the Sudanese population, with limited divergence suggesting relative isolation or stable population structure. However, the lack of available complete mtDNA sequences from neighboring African countries limits the broader phylogeographic interpretation. Future studies focusing on sequencing and analyzing mitochondrial genomes from additional African isolates are essential to enhance phylogeographic resolution and improve our understanding of regional parasite diversity. The variability observed in the D-loop region highlights its potential as a marker for studying recent evolutionary changes, though its lower resolution necessitates its use alongside more conserved regions for accurate phylogenetic inference. These findings not only enhance our understanding of *F. gigantica*’s evolutionary biology but also provide valuable genetic markers that can be leveraged for epidemiological studies, phylogeographic research, and the development of targeted control strategies, contributing significantly to global research efforts on fascioliasis.

In conclusion, the complete mitochondrial genomes of *F. gigantica* from Sudan provide valuable insights into the genetic diversity, evolutionary dynamics, and adaptation mechanisms of this parasite. These findings contribute to the broader understanding of *Fasciola* species globally and offer essential data for advancing epidemiological surveillance, phylogeographic studies, and the development of targeted control strategies against fascioliasis. The integration of both conserved and variable regions of the mitochondrial genome is crucial for a comprehensive understanding of the parasite’s evolution and adaptability. To further enhance the significance and resolution of such studies, future research should aim to incorporate nuclear genome data, comparative analyses, and functional characterization of genes to better elucidate host–parasite interactions and the evolutionary pressures shaping *F. gigantica* populations.

## Data Availability

The datasets presented in this study can be found in online repositories. The names of the repository/repositories and accession number(s) can be found at: https://www.ncbi.nlm.nih.gov/genbank/, LC649568–LC649583.

## Footnotes

^1^ http://wolfe.ucd.ie/GenomeVx/

^2^ https://www.ncbi.nlm.nih.gov/gene/17963523

## References

[1] FAO/WHO. (2014). Multicriteria-based ranking for risk Management of Food-Borne Parasites. p. 287.

[2] Mas-Coma S Bargues MD Valero MA. Fascioliasis and other plant-borne trematode zoonoses. Int J Parasitol. (2005) 35:1255–78. doi: 10.1016/j.ijpara.2005.07.010, PMID: 16150452

[3] Boore JL. Animal mitochondrial genomes. Nucleic Acids Res. (1999) 27:1767–80. doi: 10.1093/nar/27.8.1767, PMID: 10101183 PMC148383

[4] Rollinson D Kaukas A Johnston DA Simpson AJ Tanaka M. Some molecular insights into schistosome evolution. Int J Parasitol. (1997) 27:11–28. doi: 10.1016/s0020-7519(96)00169-5, PMID: 9076525

[5] Rollinson D Kaukas A Johnston DA Simpson AJ Tanaka M. Some molecular insights into schisto some evolution. Int J Parasitol. (1997) 27:11–28. PMID: 9076525 10.1016/s0020-7519(96)00169-5

[6] McNulty SN Tort JF Rinaldi G Fischer K Rosa BA Smircich P. Genomes of *Fasciola hepatica* from the Americas reveal colonization with *Neorickettsia* Endobacteria related to the agents of Potomac horse and human Sennetsu fevers. PLoS Genet. (2017) 13:e1006537. doi: 10.1371/journal.pgen.1006537, PMID: 28060841 PMC5257007

[7] Luo X Cui K Wang Z Li Z Wu Z Huang W. High-quality reference genome of *Fasciola gigantica*: insights into the genomic signatures of transposon-mediated evolution and specific parasitic adaption in tropical regions. PLoS Negl Trop Dis. (2021) 15:e0009750. doi: 10.1371/journal.pntd.0009750, PMID: 34610021 PMC8519440

[8] Gu MJ Huang WL Li YS Dong HF Zhao QP. Complete mitochondrial genomes of *Paragonimus westermani* in China and phylogenetic analysis of various geographical isolates. Zhongguo Xue Xi Chong Bing Fang Zhi Za Zhi. (2020) 32:28–35. doi: 10.16250/j.32.1374.2019238, PMID: 32185925

[9] Ibrahim K Chatanga E Mohamed NS Alasmari S Nakao R Salim B. Intra-and interspecies variation and population dynamics of *Fasciola gigantica* among ruminants in Sudan. Parasitol Res. (2024) 123:210. doi: 10.1007/s00436-024-08201-5, PMID: 38743097

[10] Shadel GS Clayton DA. Mitochondrial DNA maintenance in vertebrates. Annu Rev Biochem. (1997) 66:409–35. doi: 10.1146/annurev.biochem.66.1.409, PMID: 9242913

[11] Boore JL Brown WM. Complete DNA sequence of the mitochondrial genome of the black chiton, *Katharina tunicata*. Genetics. (1994) 138:423–43. doi: 10.1093/genetics/138.2.423, PMID: 7828825 PMC1206160

[12] Lavrov DV Brown WM. Trichinella spiralis mtDNA: a nematode mitochondrial genome that encodes a putative ATP8 and normally structured tRNAS and has a gene arrangement relatable to those of coelomate metazoans. Genetics. (2001) 157:621–37. doi: 10.1093/genetics/157.2.621, PMID: 11156984 PMC1461501

[13] Le TH Blair D McManus DP. Mitochondrial genomes of parasitic flatworms. Trends Parasitol. (2002) 18:206–13. doi: 10.1016/S1471-4922(02)02252-3, PMID: 11983601

[14] Masta SE Boore JL. The complete mitochondrial genome sequence of the spider *Habronattus oregonensis* reveals rearranged and extremely truncated tRNAs. Mol Biol Evol. (2004) 21:893–902. doi: 10.1093/molbev/msh096, PMID: 15014167

[15] Garey JR Wolstenholme DR. Platyhelminth mitochondrial DNA: evidence for early evolutionary origin of a tRNA^ser^AGN that contains a dihydrouridine arm replacement loop, and of serine-specifying AGA and AGG codons. J Mol Evol. (1989) 28:374–87. doi: 10.1007/BF02603072, PMID: 2545889

[16] Le TH Blair D McManus DP. Mitochondrial genomes of human helminths and their use as markers in population genetics and phylogeny. Acta Trop. (2000) 77:243–56. doi: 10.1016/S0001-706X(00)00157-1, PMID: 11114386

[17] Liu GH Gasser RB Young ND Song HQ Ai L Zhu XQ. Complete mitochondrial genomes of the 'intermediate form' of *Fasciola* and *Fasciola gigantica*, and their comparison with *F. hepatica*. Parasit Vectors. (2014) 7:150. doi: 10.1186/1756-3305-7-150, PMID: 24685294 PMC3997819

[18] Rambaut A. (2018). FigTree v. 1.4.4. Available online at: http://tree.bio.ed.ac.uk/software/figtree/ (Accessed August 11, 2024).

[19] Andreev DE Loughran G Fedorova AD Mikhaylova MS Shatsky IN Baranov PV. Non-AUG translation initiation in mammals. Genome Biol. (2022) 23:111. doi: 10.1186/s13059-022-02674-2 35534899 PMC9082881

[20] Quek ZBR Chang JJM Ip YCA Chan YKS Huang D. Mitogenomes reveal alternative initiation codons and lineage-specific gene order conservation in echinoderms. Mol Biol Evol. (2021) 38:981–5. doi: 10.1093/molbev/msaa262, PMID: 33027524 PMC7947835

[21] Blouin MS Yowell CA Courtney CH Dame JB. Substitution bias, rapid saturation, and the use of mtDNA for nematode systematics. Mol Biol Evol. (1998) 15:1719–27. doi: 10.1093/oxfordjournals.molbev.a025898, PMID: 9866206

[22] Cao X Slavoff SA. Non-AUG start codons: expanding and regulating the small and alternative ORFeome. Exp Cell Res. (2020) 391:111973. doi: 10.1016/j.yexcr.2020.111973, PMID: 32209305 PMC7256928

[23] Boore JL Daehler LL Brown WM. Complete sequence, gene arrangement, and genetic code of mitochondrial DNA of the cephalochordate *Branchiostoma floridae* (Amphioxus). Mol Biol Evol. (1999) 16:410–8. doi: 10.1093/oxfordjournals.molbev.a026122, PMID: 10331267

[24] Cwiklinski K Dalton JP Dufresne PJ La Course J Williams DJ Hodgkinson J. The *Fasciola hepatica* genome: gene duplication and polymorphism reveals adaptation to the host environment and the capacity for rapid evolution. Genome Biol. (2015) 16:71. doi: 10.1186/s13059-015-0632-2, PMID: 25887684 PMC4404566

[25] Le Clec'h W Chevalier FD Mattos ACA Strickland A Diaz R McDew-White M. Genetic analysis of praziquantel response in schistosome parasites implicates a transient receptor potential channel. Sci Transl Med. (2021) 13:eabj9114. doi: 10.1126/scitranslmed.abj9114, PMID: 34936381 PMC9491494

[26] Wolstenholme DR. Animal mitochondrial DNA: structure and evolution. Int Rev Cytol. (1992) 141:173–216. doi: 10.1016/s0074-7696(08)62066-5, PMID: 1452431

